# Locally Downscaled and Spatially Customizable Climate Data for Historical and Future Periods for North America

**DOI:** 10.1371/journal.pone.0156720

**Published:** 2016-06-08

**Authors:** Tongli Wang, Andreas Hamann, Dave Spittlehouse, Carlos Carroll

**Affiliations:** 1 Centre for Forest Conservation Genetics, Department of Forest and Conservation Sciences, University of British Columbia, 3041–2424 Main Mall, Vancouver, BC, V6T 1Z4, Canada; 2 Department of Renewable Resources, University of Alberta, 733 General Services Building, Edmonton, Alberta, T5H 4R1, Canada; 3 Competitiveness and Innovation Branch, Ministry of Forests, Lands and Natural Resources Operations of British Columbia, Victoria, BC, V8W 9C2, Canada; 4 Klamath Center for Conservation Research, 136 SW Washington Avenue, Suite 202, Corvallis, OR, 97333, United States of America; University of Vigo, SPAIN

## Abstract

Large volumes of gridded climate data have become available in recent years including interpolated historical data from weather stations and future predictions from general circulation models. These datasets, however, are at various spatial resolutions that need to be converted to scales meaningful for applications such as climate change risk and impact assessments or sample-based ecological research. Extracting climate data for specific locations from large datasets is not a trivial task and typically requires advanced GIS and data management skills. In this study, we developed a software package, ClimateNA, that facilitates this task and provides a user-friendly interface suitable for resource managers and decision makers as well as scientists. The software locally downscales historical and future monthly climate data layers into scale-free point estimates of climate values for the entire North American continent. The software also calculates a large number of biologically relevant climate variables that are usually derived from daily weather data. ClimateNA covers 1) 104 years of historical data (1901–2014) in monthly, annual, decadal and 30-year time steps; 2) three paleoclimatic periods (Last Glacial Maximum, Mid Holocene and Last Millennium); 3) three future periods (2020s, 2050s and 2080s); and 4) annual time-series of model projections for 2011–2100. Multiple general circulation models (GCMs) were included for both paleo and future periods, and two representative concentration pathways (RCP4.5 and 8.5) were chosen for future climate data.

## Introduction

With increasing importance of climate change related research and applications in mitigation and adaptation, the demand for accurate and accessible climate databases is high. Historical climate data are necessary for understanding the relationships between climate and biological response of organisms, or general patterns of ecological adaptations to local climate environments. Such insight can be used to build mechanistic or statistical models in ecology and other fields of study [1, 2]. Future climate projections from general circulation models can then be used to predict the potential impact of climate change and provide information for developing adaptive strategies to address a changing climate.

For ecological modeling, climate data are required to represent the climate conditions as close as possible to the locations where organisms reside [3, 4]. However, climate data generated by general circulation models (GCMs) [5] are at a coarse spatial resolution (100 ~ 300 km) and do not meet such requirements. Climate data from regional climate models (RCMs) or Global Time Series at Climate Research Unit (CRU) [6] have higher spatial resolutions (10 ~ 50 km), but they are still far too coarse to characterize climate habitat for organisms in complex landscape, such as mountainous areas. For these types of applications, climate data at moderate spatial resolutions (800 ~ 4000 m) are generated by interpolating observations from weather stations and overlaying projections from general circulation models with the delta method. WorldClim [7], for example, provides a large number of climate variables for the reference period 1950–2000 at various spatial resolutions generated using the ANUSPLIN interpolation method [8]. Another widely used database is based on the PRISM interpolation method [9], which produces climate data for the United States and some other regions using a combination of a statistical approach and an expert knowledge-based adjustment to consider rain shadows, coastal effects, and winter temperature inversions in mountainous regions. Mosier *et al*. [10] have recently generated global time series at 800 m for monthly total precipitation and mean temperature through downscaling monthly time series data from CRU, M&W and Global Precipitation Climatology Centre [11].

A general disadvantage of gridded climate databases such as WorldClim or PRISM is that they are quite large, so that extracting relevant information for a set of sample points or a local area of interest can be tedious and requires advanced skills in working with geographic information systems (GIS). Secondly, they are fundamentally limited in characterizing sample points that have inaccurate spatial information (for example, reported to the nearest minute). Such errors in spatial accuracy are particularly problematic in steep mountainous terrain, where a medium-resolution grid cell would still span climate environments with several hundred meters difference in elevation. We have addressed this problem previously with a software solution ClimateWNA [12], which builds on PRISM and ANUSPLIN data to generate scale-free climate data through a combination of bilinear interpolation and elevation adjustments. Bilinear interpolation interpolates the gridded baseline data into a seamless surface, while the elevation adjustment improves the prediction accuracy at specific locations using a digital elevation model or recorded sample elevation. Elevation adjustments are implemented by empirical lapse rates that vary among variables, location, and elevation. They are calculated as partial derivative functions with respect to elevation from polynomial functions that reflect the relationship between a monthly climate variable and latitude, longitude, elevation and their transformations and interactions [13, 14].

Our lapse rate adjustment approach is applied to 36 basic climate variables that are widely available and measured according to standardized methods at weather stations worldwide. These variables include average monthly minimum and maximum temperature and monthly precipitation. Most biological or ecological modeling applications do not rely on those primary monthly climate variables directly [15–17]. We therefore also provide 24 climatic variables that are relevant in ecology (bioclimatic variables) or that are relevant for other applications, such as infrastructure planning. Some important derived climate variables, such as growing season precipitation, can easily be calculated from monthly data, but others such as growing degree-days and frost-free period require daily climate data to calculate. Rather than including daily data in the software package, which would increase its size by two orders of magnitude, we found that it is possible to estimate variables that summarize daily data (such as growing degree days over the course of a year) from monthly data with high accuracy [12]. These algorithms are also included in the ClimateWNA software package.

Like other datasets, we make use of the delta method to overlay a medium resolution (4km) baseline dataset with historical anomaly data that are provided at about 50km resolution, and projections from GCMs that range from 100 to 300km resolution. The advantage of our approach is that the software executes these overlays on demand for sample points or local areas. Consequently, the comprehensive climate database of more than 20,000 spatial layers of historical and future data that is included with the software package remains small and can be queried instantly (or within seconds or minutes for complex queries) on a regular personal computer. Our implementation of the delta method is slightly different from others in that we replace the medium resolution baseline with scale-free data generated by ClimateWNA while the anomaly is bilinearly interpolated to match the scale-free baseline data, so that the output of ClimateWNA for historical or future period is also scale-free (i.e. not gridded but directly estimable for any location). The approach has been shown to considerably improve the statistical accuracy of climate estimates when compared to regular climate grids, when tested against original weather station data [12].

Our previous software packages have been extensively used in ecology, hydrology, forestry, agriculture, and urban planning in western North America. Notably, users include university researchers, consultants, government planners and industry managers with sizable portion of citations for data use in the “gray literature” such as government publications, extension publications, internal and consultant reports. Here, we contribute an extension of our data coverage to the entire North American continent. We also developed algorithms to improve accuracy and to extend the functionality of the new ClimateNA software package. Specifically, this release includes the following new developments: 1) a new 4km resolution baseline climate layer for 36 basic variables for the entire contiguous North American continent; 2) new dynamic local downscaling algorithms, which are broadly applicable and sensitive to local-scale variations in lapse rates; 3) additional biological relevant climate variables at monthly and seasonal scales; and 4) an integration of downscale gridded climate data for historical, paleo and future years and periods. In this paper, we also validate the effectiveness of the improved downscaling approaches and the accuracy of new climate variables against observations from weather stations.

## Data and Methods

### Baseline climate data

We used the monthly temperature and precipitation data for the normal period of 1961–1990 as a baseline data for the development of ClimateNA. The baseline dataset was compiled from several data sources listed in Table 1, which were resampled to a uniform 2.5 arcmin (approximately 4 km) resolution grid, and adjusted to 1961–1990 normal period as explained below. The original datasets were at various spatial resolutions and for two different normal periods. Unifying the data to a lower resolution (4 x 4 km) was necessary due to the size of the dataset for such a large region and has ultimately little effect on the accuracy of climate estimates because our lapse-rate based downscaling methodology is designed to recover elevation-driven spatial variation from lower resolution grids. We use the standard normal period from 1961–1990 defined by the World Meteorological Organisation (WMO) as baseline data for three reasons. First, spatial weather station coverage is the best for this period to allow for the development of a reliable interpolated baseline dataset. Second, to keep the reference period unchanged from our previous packages and to serve as the anchor for the observation of the long-time climate development. Third, the period is a useful reference in ecological research because it precedes a significant anthropogenic warming signal [18]. However, our software packages allow users to generate climate data for other normal periods including 1981–2010.

**Table 1:** Sources of climate data used to generate the baseline climate normal (1961–1990) grids for the ClimateNA software package.

Region	Data source	Spatial resolution	Period	Reference
British Columbia in Canada	PRISM*	800 x 800 m	1971–2000	[9]
Prairie provinces in Canada	PRISM	4 x 4 km	1961–1990	[29]
United States except Alaska	PRISM	800 x 800 m	1971–2000	[9]
The rest of North America (Northern and eastern Canada and Mexico)	ANUSLIN interpolated	4 x 4 km	1961–1990	[8]

* Data were provided by Faron Anslow at the Pacific Climate Impacts Consortium.

For adjusting the period from 1971–2000 to 1961–1990, we first developed anomalies for 1971–2000 relative to 1961–1990 using CRU data (described below) and bilinearly interpolated to the same spatial resolution as the target baseline dataset. The adjustment was achieved by subtracting the anomalies from the 1971–2000 normal data. The 1961–1990 dataset is a mosaic of 5 datasets covering the lower 49 states (PRISM), British Columbia and the Yukon Territories (PRISM), Alaska (PRISM), Alberta, Saskatchewan, and Manitoba below 55° latitude (PRISM), Northern and eastern Canada (WorldClim) and Mexico (WorldClim). The coverages were trimmed to an overlap of 50km, and were then merged with a gradient tool. An equal weight (50%) was allocated to each of the two overlapping datasets at the center of the overlap, and the weight was gradually reduced to 0 at the boundary of each dataset. The complete baseline dataset comprised average monthly maximum and minimum temperatures and monthly total precipitation for a total of 36 primary monthly climate variables, plus the mean elevation of each grid cell (see Table 1).

### Historical and future anomaly data

Monthly temperature and precipitation data for recent years were obtained from historical time series data generated at the Climate Research Unit (CRU) at the University of East Anglia [6]. The version incorporated in the current ClimateNA package is CRU Ts3.23 [19]. The spatial resolution of the data is 0.5 x 0.5° and covers the period of 1901–2014 (to be updated when available). The original data were developed based on anomalies relative to the reference period 1961–1990, but absolute values were subsequently derived for each individual year [6]. We recover the original anomalies by subtracting the 1961–1990 CRU average from the annual climate layers.

The monthly temperature and precipitation data for future periods were from General Circulation Models (GCMs) of the Climate Model Intercomparison Project 5 (CMIP5) corresponding to the Fifth Assessment Report of the Intergovernmental Panel for Climate Change [18]. Fifteen GCMs were selected that represent all major clusters of similar AOGCMs [20]. We included two greenhouse gas concentration trajectories or Representative Concentration Pathways (RCP): RCP 4.5 and RCP 8.5 [5]. In RCP 4.5, emissions peak in the 2040s and then decline. RCP 8.5 assumes that emissions continue to increase throughout the century. We omitted an optimistic and intermediate scenario with a peak in emissions around 2020 and 2080. When multiple runs were available for each GCM, an ensemble average was taken over the available runs (but limited to a maximum of five). We summarized annual GCM projections into 30-year time periods that we hereafter referred to as 2020s (2011–2040), 2050s (2041–2070), and 2080s (2071–2100). Time-series of annual projections were also included for the years between 2011–2100 for six of the selected 15 GCMs and the two RCPs. For the time-series, we only used the first run for each model and RCP to keep its original variability.

General circulation models are routinely tested for their realism through their hindcasting abilities. For paleoecological research, such hindcasts provide valuable climate information that we have also included. For paleoclimate, monthly data were obtained for the Last Glacial Maximum (LGM, 21,000 years ago), Mid-Holocene (6,000 years ago) and Last millennium (1,000 years ago) from four GCMs of the CMIP5. We took the averages of monthly climate projections over the first 50 years of each paleoclimatic period (essentially a point sample in a paleoclimatic context), which was the minimum period available among the four GCMs.

As GCM data are available at various spatial resolutions, ranging from 0.75 x 0.75° through 2.85 x 2.85°, we interpolated the GCM data to the resolution of 1 x 1° using bilinear interpolation for simple integration into ClimateNA. To implement the delta method just as described for overlaying the monthly historical anomalies, we also converted GCM projections to anomalies by subtracting the average of GCM projections for the 1961–1990 reference period.

### Downscaling of climate data

Instead of using the midpoint values of each grid cell to represent its entire area, we used a combination of bilinear interpolation and local elevation adjustment approaches to downscale the baseline monthly grid data (4 × 4 km) to scale-free point data. The program first uses bilinear interpolation to estimate values between midpoints of the four neighbor grids to generate a seamless surface for each monthly climate variables, avoiding step-artifacts at grid boundaries. The algorithms then retrieve monthly climate data and elevation values for a location from the corresponding grid cell plus eight surrounding cells. The climate and elevation values of the nine cells are used to calculate differences in a climate variable and in elevation between all 36 possible pairs. A simple linear regression of the differences in the climate variable on the difference in elevation is then established, and the slope of the regression is used as the empirical lapse rate for each climate variable at each specific location. As the local regressions are dynamically developed along with locations of inquiry, we call this downscaling method a “dynamic local downscaling” approach. To avoid over-adjustments due to a weak linear relationship, each lapse rate was weighted by the R-square value of the local linear regression.

Historical, paleo and future anomaly data are also downscaled prior to applying the delta method, using bilinear interpolation to create seamless surfaces. The interpolated anomalies were then added onto the downscaled baseline monthly climate normal data (scale-free) to arrive at the final climate surface at a specified resolution or for point data. With this approach, the original baseline portion (i.e., absolute values for the 1961–1990 normal period) of the historical and future climate data were replaced by the scale-free climate data generated by ClimateNA. Because elevation-adjusted baseline data generated by ClimateNA typically has much higher spatial resolution than the historical and future climate data, this process preserves the accuracies for both historical and future climate data with respect to accurately representing climate gradients that are driven by local topography (assuming that they will have similar effects in the past and future). However, the approach does not change or improve GCM anomaly projections or the historical anomalies data *per se*.

### Development of additional climate variables

The baseline dataset contains 36 primary monthly climate variables. For applications in ecology, we provide many additional biologically relevant climate variables. Many of these additional variables need to be calculated using daily climate data, which are not available in ClimateNA. We estimated these variables based on empirical or mechanistic relationships between these variables calculated using daily observations and monthly climate variables from weather stations across the entire North America. We called these variables “derived climate variables”. Some of them have been developed in previous studies for smaller regions at the annual scale [12, 13]. In this study, we developed the derived climate variables at monthly scale, then summed up to seasonal and annual scales. The steps included: 1) calculating derived climate variables for each month (e.g., degree days) from daily weather station data; 2) building relationships (or functions) between the derived climate variables and observed (or calculated) monthly climate variables; 3) applying the functions in ClimateNA to estimate derived climate variables using monthly climate variables generated by ClimateNA.

**Fig 1:**
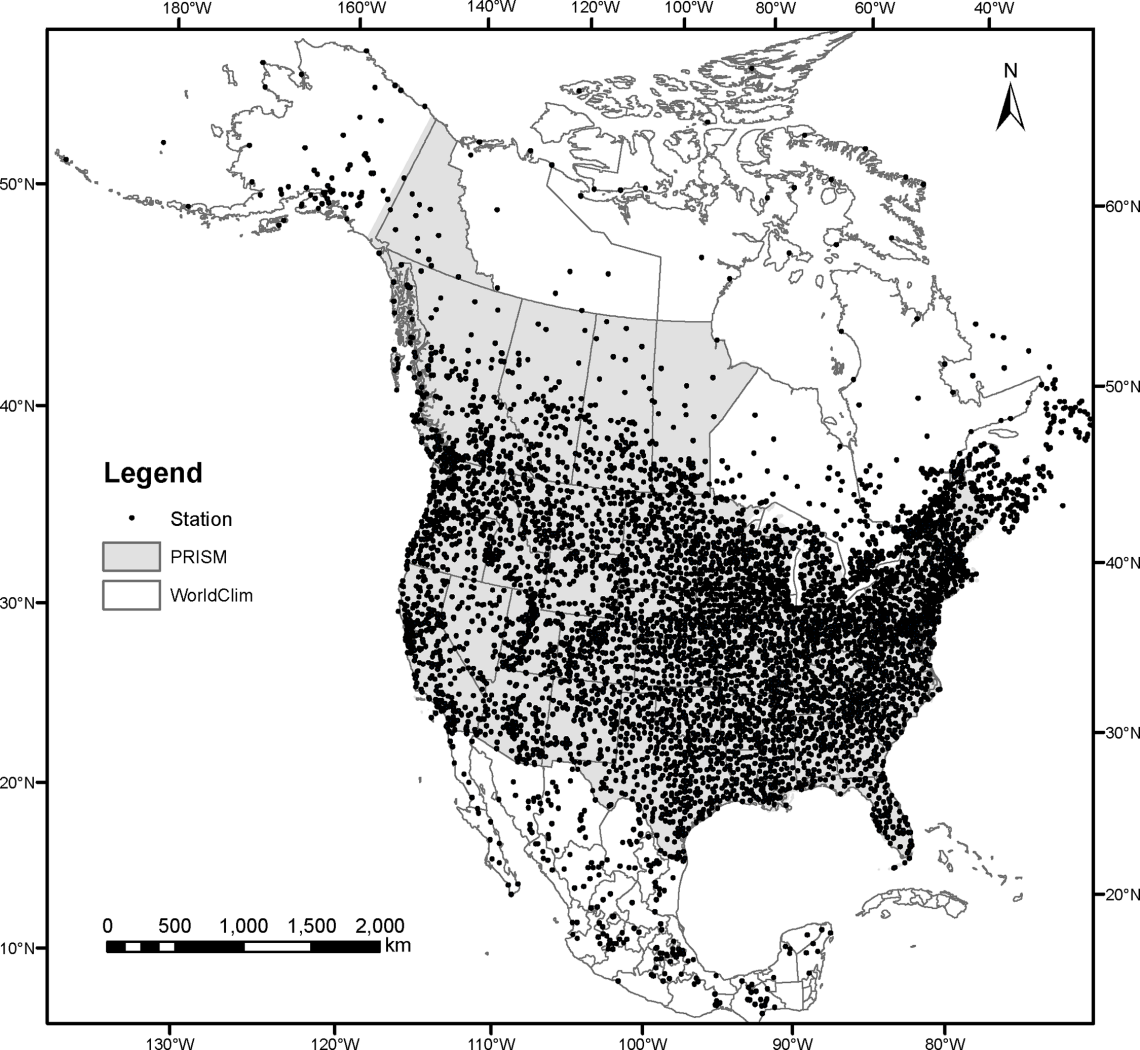
Distribution of 4891 weather stations and the baseline data sources (PRISM and WorldClim) within the coverage of ClimateNA.

Observed daily climate data were obtained from 4,891 weather stations in North America from the Daily Global Historical Climatology Network (http://www.ncdc.noaa.gov). The distribution of the weather stations is shown in Fig 1. Due to the wide range of variation in climate in North America, no single linear, polynomial or nonlinear function was found to adequately reflect the relationships between degree-days and monthly climate variables. We therefore applied piecewise functions, which combine a linear function and a nonlinear function, to model these relationships between various forms of monthly degree-day variables and monthly temperatures. The degree-day variables include degree-days below 0°C (DD < 0), degree-days above 5°C (DD>5), degree-days below 18°C (DD<18) and degree-days above 18°C (DD>18). The general form of the piecewise functions of all degree-days (*DD*_*m*_) is:

$${DD}_{m}=\{\begin{array}{l} \;if\;T_{m}>k,\;\frac{a}{1+e^{-\left(\frac{T_{m}-T_{0}}{b}\right)}}\\ \;if\;T_{m}\leq k,\;c+\beta T_{m} \end{array}$$ (1)

where, *T*_*m*_ is the monthly mean temperature for the *m* month; *k*, *a*, *b*, *T*_*0*_, *c* and *β* are the six parameters to be optimized.

For number of frost-free days (NFFD) and precipitation as snow (PAS), a sigmoid function was used to model the relationship between these monthly variables and monthly temperatures:

$${NFFD}_{m}(or\;PAS)=\frac{a}{1+e^{-\left(\frac{T_{m}-T_{0}}{b}\right)}}$$ (2)

where, *T*_*m*_ is the monthly minimum temperature for the *m* month; *a*, *b and T*_*0*_ are the three parameters to be optimized.

To estimate the length of the frost-free period (FFP), the beginning the frost-free period (bFFP) and the end of the frost-free period (eFFP), we used the same polynomial functions as ClimateWNA [12] for bFFP and eFFP while the parameters were estimated based on observations from all weather stations in North America.

For extreme minimum temperature (EMT) and extreme maximum temperature (EXT) expected over a 30-year period, polynomial functions were used as follows:

$$EMT=a+bTmin01+{cTmin01}^{2}+{dTmin12}^{2}+{eTD}^{2}$$ (3)

$$EXT=a+bTmax07+{cTmax07}^{2}+dTmax08+{eTmax08}^{2}+fTD$$ (4)

where, *a*, *b*, *c*, *d*, *e* and *f* are the parameters to be optimized; *Tmin01* and *Tmin12* are monthly minimum temperature for January and December; *Tmax07* and *Tmax08* are monthly maximum temperature for July and August, respectively; and *TD* is continentality (the difference between the mean temperatures of the warmest and coldest months).

Monthly average relative humidity (*RH* %) is calculated from the monthly maximum and minimum air temperature following [21]. Monthly reference evaporation (*Eref*_*m*_ mm) is calculated from the monthly air temperature using the Hargreaves 1985 method [12, 22]. It was evaluated against the ASCE Standardized Reference Evapotranspiration (ASCE EWRI 2005). If the monthly average air temperature is less than 0°C then *Eref*_*m*_ = 0. The monthly climatic moisture deficit (*CMD*_*m*_ mm) is 0 if *Eref*_*m*_ *< P*_*m*_, where *P*_*m*_ is the monthly precipitation (mm), otherwise

$${CMD}_{m}={Eref}_{m}-P_{m}$$ (5)

### Validation of climate variable estimates

The accuracies of the climate variables generated by ClimateNA were evaluated against observations from 4,891 weather stations shown in Fig 1. Observed monthly normals of the primary climate variables for the reference period (1961–90) were calculated based on the daily climate data from the weather stations. The prediction errors, defined as the mean absolute error (MAE), were used to evaluate the accuracy of the climate variables generated by ClimateNA for the baseline data. We also compared the prediction accuracies between ClimateNA output and the original PRISM (800 x 800 m) for areas with the PRISM data available (US and BC) against 4,257 weather stations within the coverage. It should be noted that these assessments are not truly independent validations of the original data layers, as many of these station data were included in the development of the original PRISM and WorldClim grids. Rather, the accuracy assessments evaluate the relative improvements achieved by our downscaling algorithms.

## Results

### Effects of local downscaling algorithms

**Fig 2:**
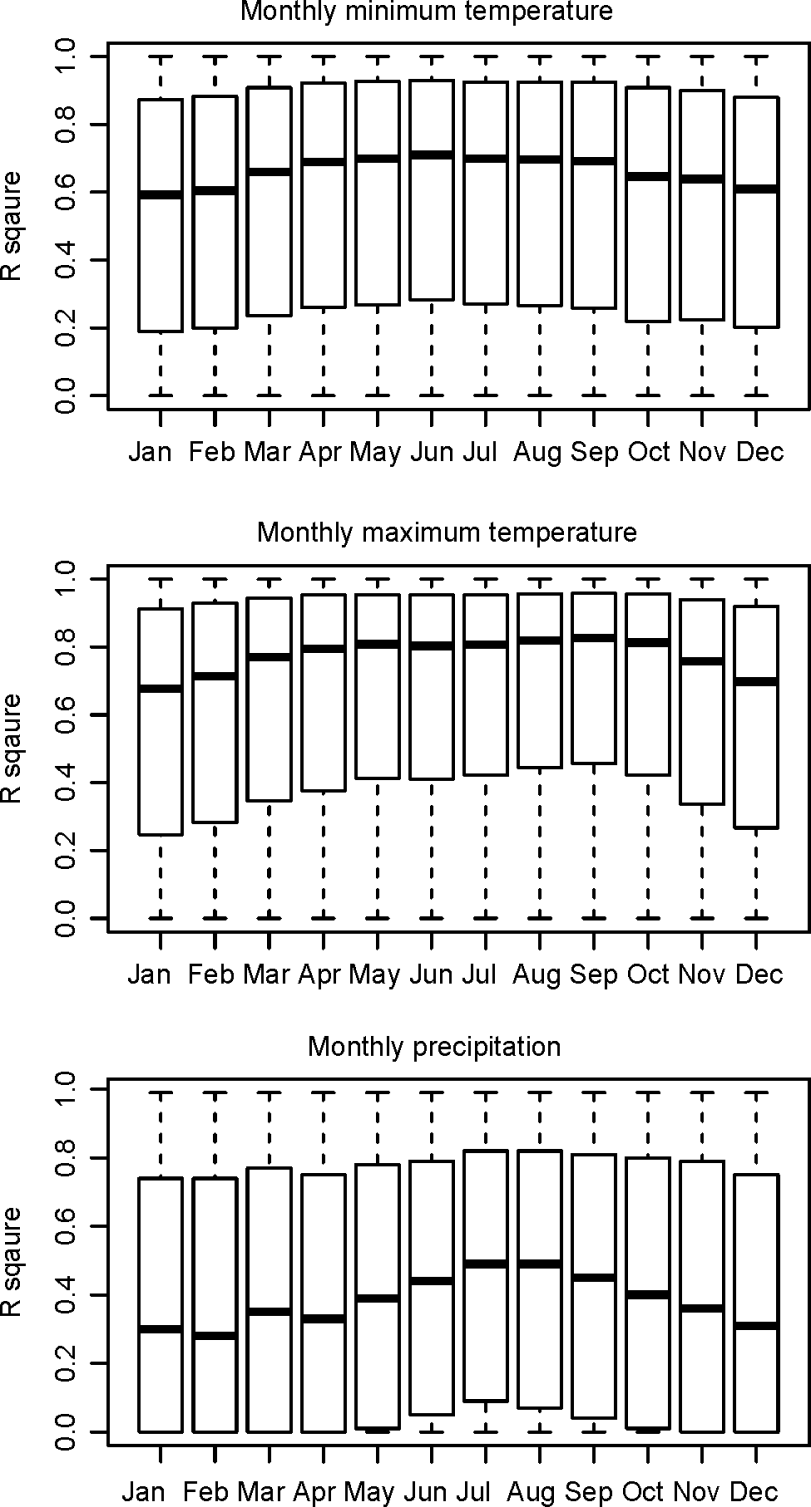
Proportions of variance explained (*R* square values) by local linear regression in total variation among the nine neighbor pixels for monthly minimum and maximum temperatures, and monthly precipitation across the entire North America. The extent of the box indicates the 25^th^ and 75^th^ percentiles. The horizontal solid lines inside the boxes indicate the medians.

**Fig 3:**
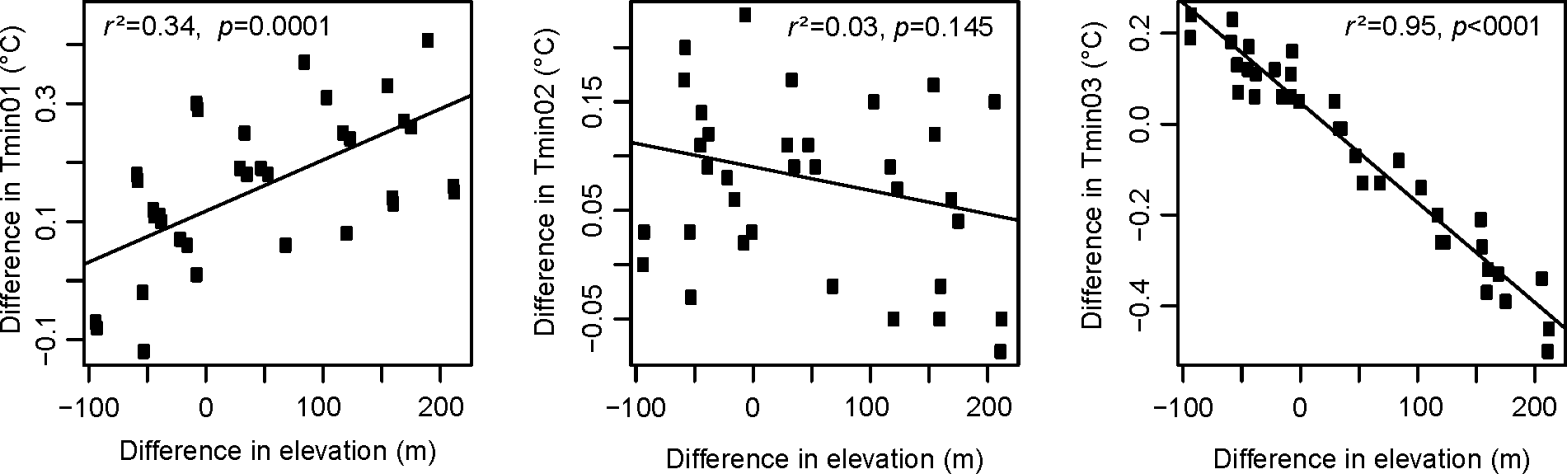
Relationships between differences in minimum temperatures in January (Tmin01), February (Tmin02) and March (Tmin03) and the change in elevation among the nine neighbor cells at a randomly picked mountain area in British Columbia (latitude = 53.88° and longitude = -125.1°).

The regressions of climate variables as a function of elevation, on which dynamic local linear downscaling approach relies, typically explained around 65% of the variance in monthly minimum temperature (Tmin), 75% in monthly maximum temperature (Tmax), and 35% in monthly precipitation (PPT) (Fig 2). The amount of variance explained varied from location to location, being greater in mountain areas, where elevation adjustment is needed, and smaller in flat areas, leading to a wide range of local R^2^ values as indicated by the box plots (Fig 2). This approach allows for high flexibility to model spatial and temporal changes of lapse rates. As an illustration of how they may vary, Fig 3 shows the changes in the lapse rate (the slope of the regression line) from January through March for a grid cell in a mountain valley, where the positive slope for Tmin01 reflects temperature inversions in mid-winter.

The dynamic lapse rate adjustment implemented by ClimateNA considerably reduced the prediction errors for monthly maximum temperatures by 5–18% (Table 2). However, the improvements for monthly minimum temperature (2–5%) and precipitation (0–3%) variables were smaller. The prediction accuracy of ClimateNA was comparable to the original PRISM (800 x 800 m), being almost identical for monthly temperatures, while the former was slightly better than the latter for monthly precipitation variables (data not shown).

**Table 2:** Comparisons in prediction standard errors between ClimateNA and the baseline climate data for primary monthly climate variables based on evaluations against observations from 4891 weather stations in North America.

	Monthly minimum temperature			Monthly maximum temperature			Monthly precipitation		
Month	Baseline (°C)	ClimateNA (°C)	Improved(%)	Observed (°C)	ClimateNA (°C)	Improved(%)	Observed (mm)	ClimateNA (mm)	Improved(%)
1	1.06	1.04	2	0.91	0.86	5	11.1	10.8	3
2	1.04	1.02	2	0.89	0.83	6	8.3	8.2	1
3	0.89	0.86	3	0.86	0.78	10	8.7	8.5	2
4	0.78	0.74	5	0.78	0.65	16	9.1	9.1	1
5	0.77	0.74	4	0.76	0.64	16	7.6	7.5	0
6	0.81	0.78	4	0.77	0.64	17	7.8	7.8	1
7	0.82	0.80	3	0.73	0.61	17	7.9	7.9	1
8	0.84	0.82	3	0.75	0.62	18	7.8	7.8	0
9	0.87	0.84	3	0.74	0.61	17	12.9	12.9	0
10	0.90	0.87	3	0.72	0.61	16	9.3	9.3	0
11	0.81	0.79	3	0.70	0.62	11	9.5	9.4	1
12	0.96	0.93	3	0.74	0.69	7	10.5	10.2	3
Average	0.88	0.85	3	0.78	0.68	13	9.2	9.1	1

### Accuracy of derived climate variables

**Fig 4:**
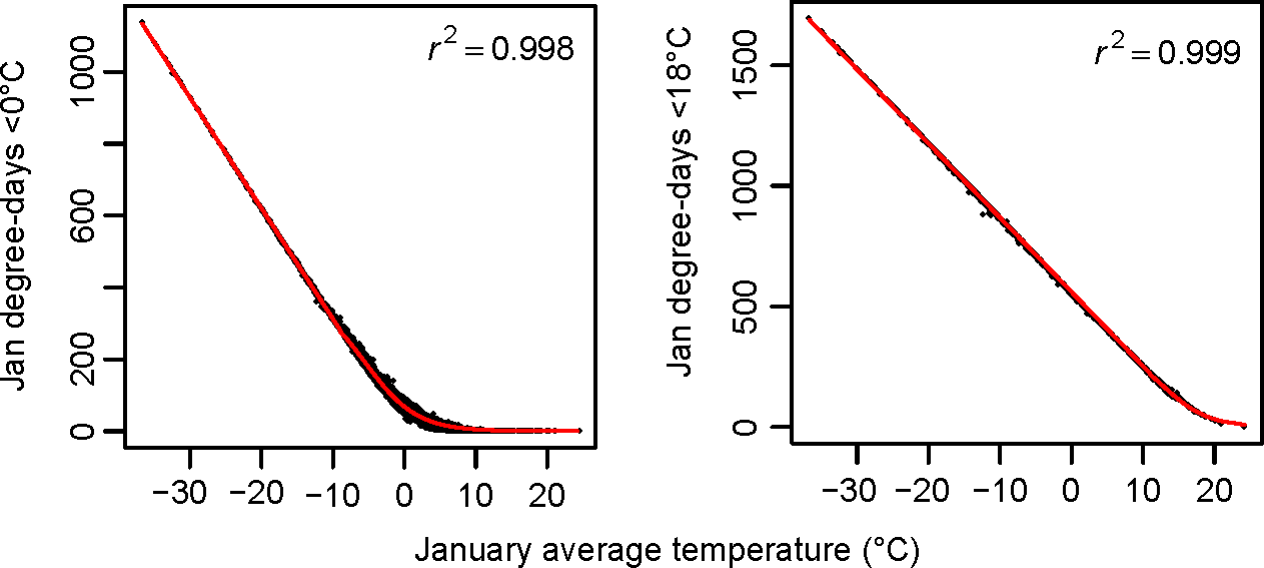
Illustrations of the model fit (red line) of the piecewise function (linear and nonlinear) for monthly degree-days DD<0 and DD<18 on monthly mean temperature for January for the base line period 1961–1990 for 4891 locations (black points).

**Fig 5:**
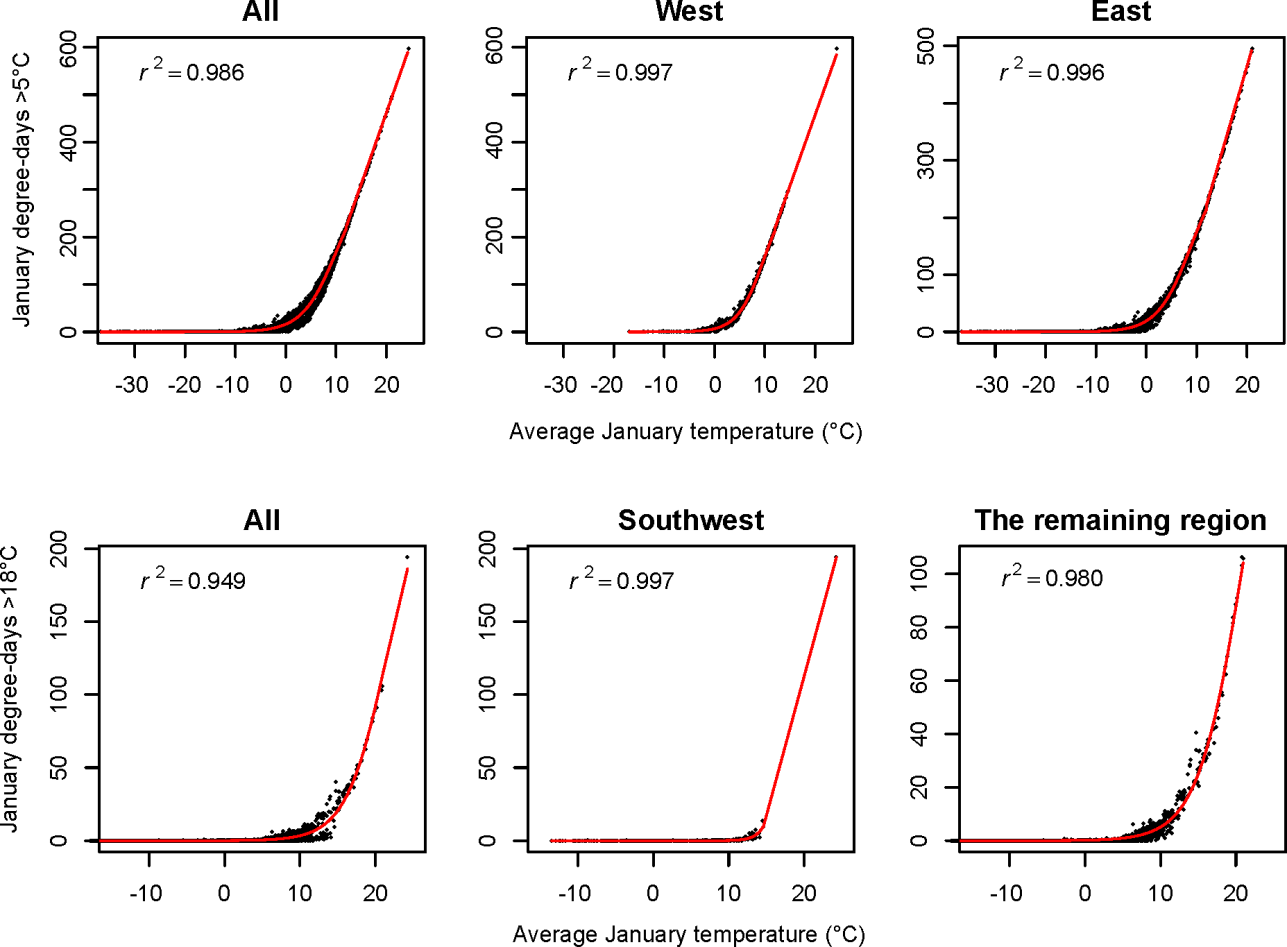
Illustrations of the model fit (red line) of the piecewise function (linear and nonlinear) with all (4891) and split samples for monthly degree-days DD>5 and DD>18 on monthly mean temperature for January for the base line period 1961–1990.

The relationships between monthly data and biologically relevant climate variables derived from daily weather station data could generally be well described by the piecewise functions or nonlinear functions (Figs 4 and 5). However, the relationships showed distinct patterns between the west and the east for growing degree days (DD>5°C) and between the southwest and the rest for DD>18°C. Thus, the functions for these two variables were built separately by region to better capture relationships as illustrated in Fig 5.

The amount of variance explained and the prediction errors for all biologically relevant climate variables derived from observed daily climate data are listed in Table 3. The functions (S1 File) and their parameters as well as the results of evaluations (S1–S6 Tables) are listed in the Supporting Information. In most cases, the predictive accuracy of monthly variables for derived variables is very high with R^2^ values larger than 0.95 (S1–S5 Tables). We expect that accuracies for derived variables that are derived for individual months is somewhat lower than for annual derived variables, and that is generally confirmed (note that the annual MAEs must be divided by 12 to be directly comparable with the monthly MAEs). Our lowest R^2^ values are for precipitation as snow (PAS) at a monthly time scale (S6 Table).

**Table 3:** The amount of variance in observed climate variables explained by ClimateNA derived variables and their prediction standard errors.

Variable	Variance explained (%)		Prediction standard error	
	Monthly	Annual	Monthly	Annual
DD<0 (°C)	99.1–99.8	99.7	0.1–8.3	36.1
DD>5 (°C)	99.6–100.0	99.9	1.7–7.0	14.61
DD<18 (°C)	99.4–100.0	99.9	2.9–6.0	25.6
DD>18 (°C)	99.2–99.8	99.9	0.7–4.0	11.8
NFFD (day)	92.3–99.3	99.8	0.2–0.9	2.3
PAS (mm)	64.2–87.7	87.8	0.2–5.6	16.5
bFFP		96.9		5.0
eFFP		95.1		5.2
FFP		96.7		9.4
EMT (°C)		90.5		2.7
EXT (°C)		84.7		1.3

## Discussion

### Benefits of local downscaling

Elevation is the dominant factor affecting temperature at a local scale, but the effect of elevation on temperature and precipitation varies with latitude, local terrain and other factors. Temperature inversions (i.e., temperature is cooler at lower elevations) can also occur in some areas, typically in winter months. Therefore, it remains a challenge to develop global functions that accurately reflect lapse rates across a large region [12]. We found that the dynamic local regression approach developed in this study is effective for monthly temperature and precipitation variables in downscaling the baseline climate data at a moderate spatial resolution. In most cases, over 60% of the total variation in temperature among the nine neighbor tiles of the baseline climate can be explained by elevation, and it is more effective in mountain areas (>90%) where elevation adjustment is more critical than in flat areas. This approach is able to capture the elevation effect in case of temperature inversion. The amount of variation explained by elevation in monthly precipitation is smaller (~30% for most cases, >40% in mountain areas), and elevation adjustment is not possible at all using the partial derivative functions used in ClimateWNA (up to version 4.85), so the dynamic downscaling still provides a significant improvement for precipitation over previous versions of the software.

### Bioclimatic variables

New derived climate variables are an important addition to the ClimateNA software package. Climate-driven ecological and hydrological models require driving variables that represent periods longer than a month, or derived climate variables that represent summaries of daily data over a longer period. These variables are usually referred to as bioclimatic variables and are widely used in ecological modeling. For example, the most important climate variable that separating forest ecosystems in British Columbia is Continentality (TD) [23]. Precipitation as snow (PAS) and summer heat-moisture index (SMH) have been the most effective climate variables to delineate the hybrid index for hybrids between white and Engelmann spruces [16]. Various forms of degree-days, frost-free days and moisture deficit index are also often found to play important biological [24, 25], ecological [1, 26] and hydrological [27] roles. ClimateNA, with added derived climate variables at monthly and seasonal levels, will provide more options for modellers to test, identify, and make use of climate variables that closely represent drivers of ecological processes.

### Applications and limitations

**Fig 6:**
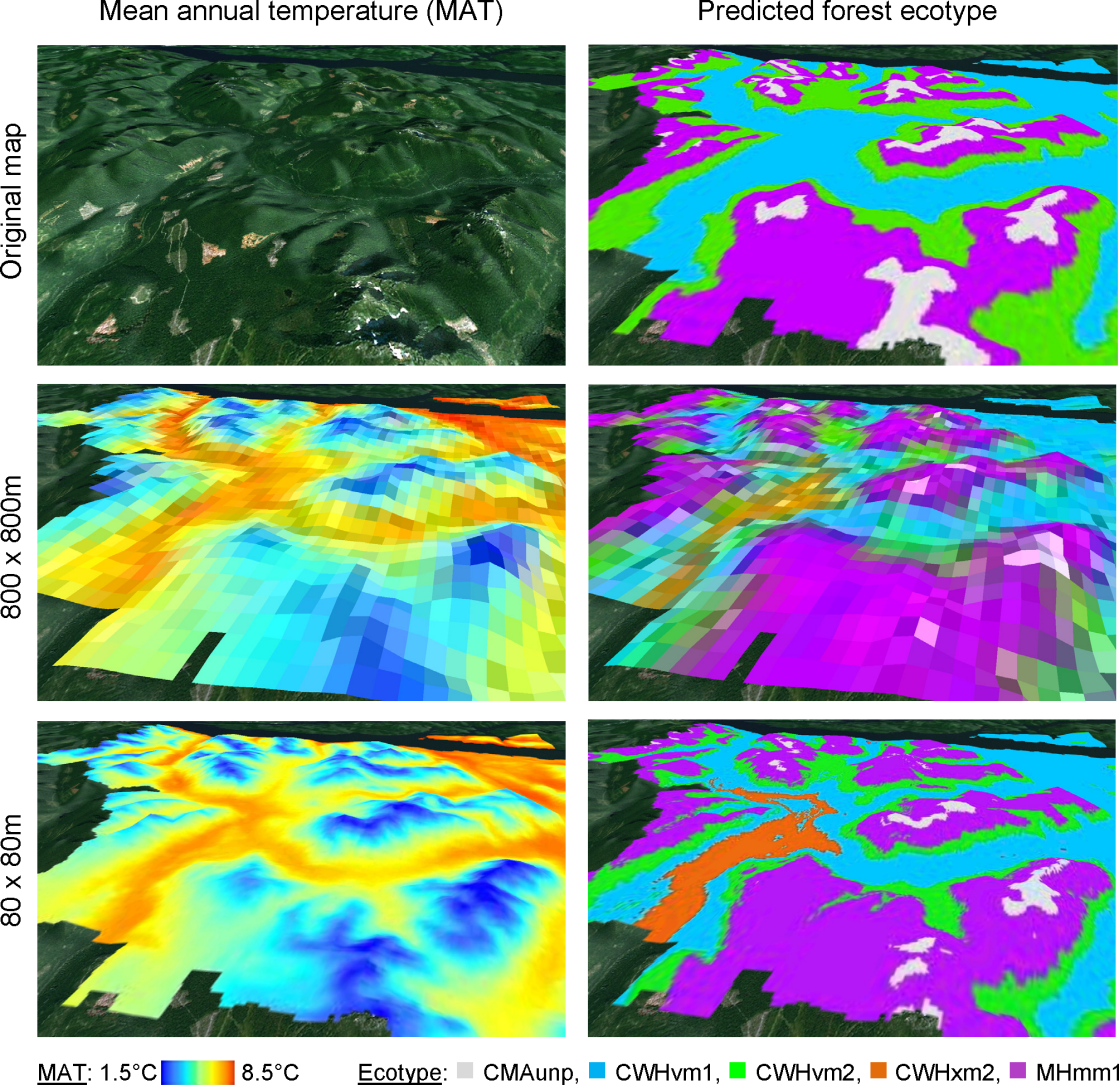
Landscape image (a) and original forest ecotype map (b) at the north end of Vancouver Island, BC, Canada (centered at Lat. = 50.362° and Long. = -126.385°). Spatial distributions of mean annual temperature (MAT) (c and e) and predicted forest ecotypes (d and f) at the spatial resolutions of 800 x 800 m (c and d) and 80 x 80 m (e and f), respectively.

ClimateNA makes it possible to generate climate data at any desirable spatial resolution. To give an example for the need of downscaling in ecological research, we visualize how climate surfaces match the topography (Fig 6*A*, 6*C* and 6*E*), and how well the predicted forest ecotypes match the originally mapped ecosystem classifications (Fig 6*B*, 6*D* and 6*F*) at different spatial resolutions (800 x 800 m and 80 x 80 m). For predicting forest ecotypes, we used ClimateNA to generate climate variables for a bioclimate envelope model of forest ecosystems in British Columbia [23]. Local climate gradients in topographically complex terrain can be a driver of forest ecotypes at very fine spatial resolutions that the ClimateNA software package can deliver (here, 80 x 80 m). For the predicted ecotypes in particular, the finer spatial resolution is critical at a forest management scale (Fig 6*B*, 6*D* and 6*F*). The coarse resolution is not able to represent the spatial distributions of the original ecotypes at the local scale.

Notwithstanding the need for high-resolution climate grids, we should also point out the general limitations of the data we provide through the software package and its underlying databases. Although climate data can be generated at very fine resolution as shown in the above example, the data are ultimately based on interpolated data from standardized weather stations, and therefore cannot include micro site effects that are driven by vegetation, water bodies, or other micro-scale physiographic features.

While the dynamic lapse-rate algorithms considerably improve on regular gridded climate data, the accuracy of the climate estimates also vary with the time period the variable represents and the variable type. As shown in the results, errors tend to be largest in complex derived variables that are not averaged over longer time periods. For example, measurements of the degree days for an individual month of an individual year come with larger uncertainties than a degree day estimate that is made for the entire year or estimates for a month that are averaged over a 30-year normal period.

Lastly, we expect variable estimates with larger errors in the remote areas where the number of weather stations is limited as shown in Fig 1. Because our validations are not independent (i.e. the weather stations we use for validation here have likely also been used by the PRISM and WorldClim groups for developing the baseline data), the average prediction error was quite small with an average of 0.77°C for monthly temperatures. However, this does not necessarily guarantee that estimates for areas without nearby weather stations have this level of precision.

### Data access

Three options were developed for accessing the downscaled climate data generated by the algorisms implemented in ClimateNA: 1) ClimateNA desktop package; 2) ClimateNA Map; and 3) a GIS raster data portal.

ClimateNA desktop package has an interactive interface for single-location and multiple-location process. For a single location, users input latitude, longitude and elevation (optional) to obtain all climate variables for a selected period (a historical, a paleo, the reference or a future period). For multiple locations, users prepare an input dataset of columns of two location IDs, latitude, longitude and elevation. There is no limit for the number of locations. A time-series function is available for users to obtain climate data for multiple years and for multiple locations. This can save a tremendous amount of time. The output file is in comma-delimited format (.CSV) and can be directly imported to ArcGIS, R, SAS or other package for data analysis or conversion to gridded data formats. The desktop version can be downloaded for free at *http://cfcg.forestry.ubc.ca/projects/climate-data/climatebcwna/#ClimateNA*

ClimateNA Map is a web version integrated to Google Maps through Google Maps APIs. It allows users to obtain the coordinates and elevation for the location of interest simply by clicking at the location on the map. The program automatically imports this information from the Google Maps to ClimateNA. The users can then click on the “Calculate” button to get all climate variables for a selected period. The output can be saved on a local computer. The map version also facilitates spatial visualization of major climate variables overlaid onto the Google Maps, which can also be downloaded as raster layers to a local computer. The map version can be accessed at *http://climatewna.com/climatena_map*

Finally, we provide gridded raster data layers that were generated for the entire North America at the spatial resolution of 1000 x 1000 meter for the normal periods 1961–1990 and 1981–2010, and three future periods 2020s, 2050s and 2080s for 8 selected CMIP5 models and two greenhouse gas emission scenarios (RCP4.5 and RCP8.5). These datasets can be downloaded at *http*:*//tinyurl*.*com/ClimateNA* or *http*:*//adaptwest*.*databasin*.*org/pages/adaptwest-climatena*. The variables can also be displayed graphically, as summarized by watershed across North America, using an interactive online data viewer (*http*:*//adaptwest*.*databasin*.*org/app/watershed-climate-explorer*). Additional variables derived from ClimateNA output, such as climate velocity [28], can also be downloaded from the portal (*http*:*//adaptwest*.*databasin*.*org/pages/adaptwest-velocitywna*).

## Conclusion

In summary, we found that the dynamic local-regression approach implemented in ClimateNA can effectively downscale the gridded baseline data into scale-free point data with improved prediction accuracy for all climate variables. The software package facilitates access to climate data at both large scales (e.g. for continental species ranges and regional ecosystem characterization and modeling), but also at local management unit scales (e.g. to climatically characterize plot and sample data). Additions of derived biologically and ecologically relevant climate variables at monthly and seasonal scales provide more options for modellers to improve their biological, ecological and hydrological models. Integration of historical, future and paleo climate data through the delta downscaling method provides convenience through integrating many climate data sources in one package. Time-series functions, interactive map-based interface and raster data portals may save users the considerable effort of processing large volume of climate data. We hope that ClimateNA will serve as a useful tool in climate related research and applications across North America under changing climate.

## Supporting Information

S1 File Equations of derived climate variables. (PDF)

S1 Table Parameters and the results of the model fit for monthly Degree-days below 0°C (DD < 0). (PDF)

S2 Table Parameters and the results of the model fit for the piecewise function for monthly Degree-days above 5°C (DD > 5). (PDF)

S3 Table Parameters and the results of the model fit for the piecewise function for monthly Degree-days below 18°C (DD < 18). (PDF)

S4 Table Parameters and the results of the model fit for the piecewise function for monthly Degree-days above 18°C (DD > 18). (PDF)

S5 Table Parameters and the results of the model fit for monthly Number of frost-free days (NFFD). (PDF)

S6 Table Parameters and the results of the model fit for monthly precipitation as snow (PAS). (PDF)

## References

[1] Rehfeldt GE, Crookston NL, Warwell MV, Evans JS. Empirical analyses of plant-climate relationships for the western United States. International Journal of Plant Sciences. 2006;167(6):1123–50. 10.1086/507711 .

[2] MacDonald RJ, Boon S, Byrne JM, Robinson MD, Rasmussen JB. Potential future climate effects on mountain hydrology, stream temperature, and native salmonid life history. Canadian Journal of Fisheries and Aquatic Sciences. 2014;71(2):189–202. 10.1139/cjfas-2013-0221 .

[3] Hamann A, Wang TL. Potential effects of climate change on ecosystem and tree species distribution in British Columbia. Ecology. 2006;87(11):2773–86. 10.1890/0012-9658(2006)87[2773:peocco]2.0.co;2 . 17168022

[4] Rehfeldt GE, Crookston NL, Saenz-Romero C, Campbell EM. North American vegetation model for land-use planning in a changing climate: a solution to large classification problems. Ecol Appl. 2012;22(1):119–41. . 22471079 10.1890/11-0495.1

[5] Taylor KE, Stouffer RJ, Meehl GA. An overview of CMIP5 and the experiment design. Bull Am Meteorol Soc. 2012;93(4):485–98. 10.1175/bams-d-11-00094.1 .

[6] Mitchell TD, Jones PD. An improved method of constructing a database of monthly climate observations and associated high-resolution grids. Int J Climatol. 2005;25(6):693–712. 10.1002/joc.1181 .

[7] Hijmans RJ, Cameron SE, Parra JL, Jones PG, Jarvis A. Very high resolution interpolated climate surfaces for global land areas. Int J Climatol. 2005;25(15):1965–78. 10.1002/joc.1276 .

[8] Hutchinson MF. A new objective method for spatial interpolation of meteorological variables from irregular networks applied to the estimation of monthly mean solar radiation, temperature, precipitation and windrun. CSIRO Division of Water Resources Tech. Memo. 89/5:95–104. 1989.

[9] Daly C, Halbleib M, Smith JI, Gibson WP, Doggett MK, Taylor GH, et al Physiographically sensitive mapping of climatological temperature and precipitation across the conterminous United States. Int J Climatol. 2008;28(15):2031–64. 10.1002/joc.1688 .

[10] Mosier TM, Hill DF, Sharp KV. 30-Arcsecond monthly climate surfaces with global land coverage. Int J Climatol. 2014;34(7):2175–88. 10.1002/joc.3829

[11] Becker A, Finger P, Meyer-Christoffer A, Rudolf B, Schamm K, Schneider U, et al A description of the global land-surface precipitation data products of the Global Precipitation Climatology Centre with sample applications including centennial (trend) analysis from 1901-present. Earth System Science Data. 2013;5(1):71–99. 10.5194/essd-5-71-2013 .

[12] Wang TL, Hamann A, Spittlehouse DL, Murdock TQ. ClimateWNA-High-Resolution Spatial Climate Data for Western North America. Journal of Applied Meteorology and Climatology. 2012;51(1):16–29. 10.1175/jamc-d-11-043.1 .

[13] Wang T, Hamann A, Spittlehouse DL, Aitken SN. Development of scale-free climate data for western Canada for use in resource management. Intl J Climatology. 2006;26(3):383–97.

[14] Hamann A, Wang TL. Models of climatic normals for genecology and climate change studies in British Columbia. Agric For Meteorol. 2005;128(3–4):211–21. 10.1016/j.agrformet.2004.10.004 .

[15] Bradley St. Clair J, Kilkenny FF, Johnson RC, Shaw NL, Weaver G. Genetic variation in adaptive traits and seed transfer zones for Pseudoroegneria spicata (bluebunch wheatgrass) in the northwestern United States. Evolutionary Applications. 2013;6(6):933–48. 10.1111/eva.12077 24062802 PMC3779094

[16] De La Torre AR, Wang TL, Jaquish B, Aitken SN. Adaptation and exogenous selection in a Picea glauca x Picea engelmannii hybrid zone: implications for forest management under climate change. New Phytol. 2014;201(2):687–99. 10.1111/nph.12540 . 24200028 PMC4285121

[17] Cortini F, Comeau PG, Bokalo M. Trembling aspen competition and climate effects on white spruce growth in boreal mixtures of Western Canada. For Ecol Manage. 2012;277:67–73. 10.1016/j.foreco.2012.04.022.

[18] IPCC. Climate Change 2014: The Physical Science Basis Contribution of Working Group I to the Fifth Assessment Report of the Intergovernmental Panel on Climate Change. Cambridge, United Kingdom and New York, NY, USA: Cambridge University Press; 2014.

[19] Harris I, Jones PD, Osborn TJ, Lister DH. Updated high-resolution grids of monthly climatic observations—the CRU TS3.10 Dataset. Int J Climatol. 2014;34(3):623–42. 10.1002/joc.3711 .

[20] Knutti R, Masson D, Gettelman A. Climate model genealogy: Generation CMIP5 and how we got there. Geophys Res Lett. 2013;40:1194–9.

[21] EWRI ASCE. The ASCE Standardized Reference Evapotranspiration Equation.: Environmental and Water Resources Institute (EWRI) of the American Society of Civil Engineers Task Committee on Standardization of Reference Evapotranspiration Calculation, ASCE, Washington, DC 2005.

[22] Hargreaves G, Allen R. History and Evaluation of Hargreaves Evapotranspiration Equation. Journal of Irrigation and Drainage Engineering. 2003;129(1):53–63. 10.1061/(ASCE)0733-9437(2003)129:1(53)

[23] Wang TL, Campbell EM, O'Neill GA, Aitken SN. Projecting future distributions of ecosystem climate niches: Uncertainties and management applications. For Ecol Manage. 2012;279:128–40. 10.1016/j.foreco.2012.05.034 .

[24] Rehfeldt GE, Wykoff WR, Ying CC. Physiologic plasticity, evolution, and impacts of a changing climate on Pinus contorta. Clim Chang. 2001;50(3):355–76.

[25] Liu Y, Wang T, EI-Kassaby YA. Contributions of dynamic environmental signals during life-cycle transitions to early life-history traits in lodgepole pine (*Pinus contorta* Dougl.). Biogeosciences Discuss. 2015;12:14105–38.

[26] Fitterer JL, Nelson TA, Coops NC, Wulder MA, Mahony NA. Exploring the ecological processes driving geographical patterns of breeding bird richness in British Columbia, Canada. Ecol Appl. 2013;23(4):888–903. . 23865238 10.1890/12-1225.1

[27] Shrestha RR. Modelling spatial and temporal variability of hydrologic impacts of climate change in the Fraser River basin, British Columbia, Canada HYDROLOGIC IMPACTS OF CLIMATE CHANGE IN THE FRASER RIVER BASIN. Hydrol Process. 2012;26(12):1840–60. 10.1002/hyp.9283

[28] Hamann A, Roberts DR, Barber QE, Carroll C, Nielsen SE. Velocity of climate change algorithms for guiding conservation and management. Global Change Biology. 2015;21(2):997–1004. 10.1111/gcb.12736 . 25310933

[29] Daly C, Gibson WP, Taylor GH, Johnson GL, Pasteris P. A knowledge-based approach to the statistical mapping of climate. Clim Res. 2002;22(2):99–113. 10.3354/cr022099 .

