# Supplementary material for: Locally Downscaled and Spatially Customizable Climate Data for Historical and Future Periods for North America
Source: PLoS One. 2016 Jun 8;11(6):e0156720. doi: 10.1371/journal.pone.0156720 (PMC4898765; doi:10.1371/journal.pone.0156720)
Supplement: S1 File — (PDF) [file pone.0156720.s001.pdf]

S1 File. Equations of derived climate variables

The general piecewise function for degree-day variables:

$$DD_m = \begin{cases} if\ T_m > k, & \frac{a}{1+e^{-\left(\frac{T_m - T_0}{b}\right)}} \\ if\ T_m \le k, & c + \beta T_m \end{cases}$$

Where, T_m is the monthly mean temperature for the *m* month; *k, a, b,* T_0*, c* and *β* are the six parameters to be optimized. This general piecewise function applies to degree-days below 0°C (DD < 0), degree-days above 5°C (DD > 5), degree-days below 18°C (DD < 18) and degree-days above 18°C (DD > 18). Estimated parameters and results of model fit can be found in S1 – S4 Tables.

Number of frost-free days (NFFD)

The general function for monthly NFFD ($NFFD_m$) is:

$$NFFD_m = \frac{a}{1+e^{-\left(\frac{T_m - T_0}{b}\right)}}$$

where, T_m is the monthly minimum temperature for the *m* month; *a, b and* T_0 are the three parameters to be optimized. Estimated parameters and results of model fit can be found in S5 Table.

Frost-free period (FFP), the day of the year on which FFP begins (bFFP) and the day of the year on which FFP ends (eFFP)

bFFP = 352.1358994 + -0.021715653 * Tmin(4)^2 + -3.542187618 * Tmin(6) + 0.020359471 * Tmin(6)^2 - 4.897998097 * TD + 0.033521327 * TD^2 - 2.164862277 *

NFFD + 0.006767633 * $NFFD^2$ - 0.00000929 * $NFFD^3$ + 0.043516586 * (TD * NFFD) - 0.00000253 * $(TD * NFFD)^2$

eFFP = 243.7752209 + 4.134210825 * Tmin(9) - 0.162876448 * $Tmin(9)^2$ + 1.248649021 * Tmin(10) + 0.145073612 * $Tmin(10)^2$ + 0.004319892 * Tmin(11) + -0.005753127 * $Tmin(11)^2$ - 0.06296471 * NFFD + 0.000399177 * $NFFD^2$

FFP = eFFP – bFFP

where Tmin is the monthly minimum temperature, TD is difference between the mean warmest monthly temperature and the mean coldest monthly temperature, and NFFD is the number of frost-free days.

Precipitation as snow (PAS)

The general function for monthly PAS (PAS_m) is

$$PAS_m = \frac{1}{1+e^{-\left(\frac{T_m - T_0}{b}\right)}}$$

Where, T_m is the monthly minimum temperature for the *m* month; *b and* T_0 are the three parameters to be optimized. Estimated parameters and results of model fit can be found in S6 Table.

Extreme minimum temperature (EMT) and extreme maximum temperature (EXT)

EMT = -23.02164 + 0.77908 * Tmin(1) + 0.67048 * Tmin(12) + 0.01075 * $TminX^2$ + 0.11565 * TD

EXT = 10.64245 + -1.92005 * Tmax(7) + 0.04816 * $Tmax(7)^2$ + 2.51176 * Tmax(8) - 0.03088 * $Tmax(8)^2$ - 0.01311 * $TmaxX^2$ + 0.33167 * TD - 0.001 * TD^2

where Tmin is the monthly minimum temperature, Tmax is the monthly maximum temperature, TmaxX is the maximum Tmax over the year, and TD is difference between the mean warmest monthly temperature and the mean coldest monthly temperature.

Relative humidity (RH)

Monthly average relative humidity (RH %) is calculated from the monthly maximum and minimum air temperature following [21]:

$$RH = 100 * es(Tmin)/es(avg)$$

$$es(avg) = [es(Tmin) + es(Tmax)]/2$$

where es(Tmin) and es(Tmax) are the saturated vapour pressure (kPa) at the monthly mean minimum and maximum air temperature (°C), respectively, and es(avg) is the monthly average saturation vapour pressure (kPa). The Teten's equation is used to calculate the saturated vapour pressure (SVP(T) kPa) as a function of temperature (T °C).

$$SVP(T)\ (kPa) = 0.6105 * \exp([17.273 * T]/[T + 237.3])$$

$$\text{For } T \Rightarrow 0°C \quad es(T) = SVP(T)$$

$$\text{For } T < 0°C \quad es(T) = SVP(T) * (1 + [T * 0.01])$$

This method will slightly overestimate the daily average relative humidity in dry environments where the nighttime relative humidity does not approach 100%.
