# Supplementary material for: Locally Downscaled and Spatially Customizable Climate Data for Historical and Future Periods for North America
Source: PLoS One. 2016 Jun 8;11(6):e0156720. doi: 10.1371/journal.pone.0156720 (PMC4898765; doi:10.1371/journal.pone.0156720)
Supplement: S1 Table — (PDF) [file pone.0156720.s002.pdf]

S1 Table. Parameters and the results of the model fit for monthly Degree-days below 0°C (DD < 0).

Month	k	a	b	T_0	β	c	Sigma	R^2
1	-9	381.2279	-5.4630	-3.59	-30.7664	5	8.4	0.998
2	-10	357.1408	-5.6865	-3.60	-27.7799	5	7.9	0.996
3	-9.5	358.7336	-4.9692	-3.31	-30.8261	5	7.9	0.993
4	-7	274.6697	-3.5915	-2.68	-29.5144	10	4.5	0.992
5	-5	169.7686	-1.2282	-1.85	-29.0289	20	1.2	0.997
6		167.0972	-2.1215	-1.95			0.5	0.958
7*		134.2072	-1.5400	-1.75				
8		101.3172	-0.9553	-1.56			0.2	0.899
9	-4.5	262.5633	-3.9104	-2.33	-32.6652	-20	0.5	0.997
10	-6.5	246.0955	-2.8570	-2.47	-30.2925	10	2.2	0.994
11	-7.5	280.4533	-3.5609	-2.84	-29.5002	10	6.1	0.993
12	-10	403.6482	-5.9513	-3.60	-30.7232	5	7.6	0.997

* Averaged between June and August as no parameters estimated.

Degree-days above 5°C (DD5)

Table S2. Parameters and the results of the model fit for the piecewise function for monthly Degree-days above 5°C.

Region	Month	k	a	b	T_0	β	c	Sigma	R^2
All	1	12	337.5699	10.0241	3.36	30.0966	-140	7.6	0.986
	2	12	302.8633	10.0060	3.31	28.0429	-140	7.5	0.988
	3	12	363.8180	10.5024	3.44	30.1222	-140	10.3	0.991
	4	12	339.8059	10.3516	3.26	29.4187	-140	7.4	0.997
	5*	12	327.1587	10.0530	2.79	30.1473	-140	3.6	0.999
	6*	13	370.5585	11.1296	3.13	29.9647	-150	2.2	1.000
	7*	15	410.0218	11.6278	3.12	30.7456	-150	1.7	1.000
	8*	15	412.2794	11.6613	3.13	30.7429	-150	1.6	1.000
	9*	13	342.8546	10.5144	2.96	29.7243	-145	2.1	1.000
	10*	12	344.9987	10.2648	3.19	30.4110	-145	5.0	0.999
	11	11	304.7169	9.5882	3.15	29.4263	-140	7.0	0.995
	12	12	341.0866	10.0869	3.29	30.1269	-140	7.0	0.990
West	1	12	299.5106	9.6754	2.58	29.8768	-140	4.3	0.997
	2	12	250.6012	9.2207	2.42	27.9937	-140	5.0	0.997
	3	12	318.4976	10.0133	2.63	30.0353	-140	6.3	0.997
	4	12	308.1068	10.0143	2.75	29.3949	-140	5.9	0.998
	11	12	322.3119	10.2178	2.85	29.3400	-140	5.0	0.998
	12	12	289.4347	9.4137	2.55	30.0054	-140	4.9	0.996
East	1	12	295.5816	8.7948	3.29	30.1798	-140	4.0	0.996
	2	12	283.2021	9.3056	3.44	28.0894	-140	3.9	0.996
	3	12	308.5948	9.1350	3.34	30.1602	-140	6.5	0.996
	4	12	309.8332	9.6150	3.13	29.4255	-140	5.9	0.998
	11	12	309.4319	9.5599	3.26	29.4366	-140	5.0	0.997
	12	12	326.1452	9.5931	3.39	30.1914	-140	3.8	0.997

* Region specific model is not necessary.

Degree-days below 18°C (DD_18)

Table S3. Parameters and the results of the model fit for the piecewise function for monthly Degree-days below 18°C.

Month	k	a	b	T_0	β	c	Sigma	R^2
1	11	342.1497	12.7839	-3.19	-30.7428	560	2.9	1.000
2	11	344.9851	12.0296	-3.29	-28.0059	500	2.9	1.000
3	11	325.8230	13.1044	-2.99	-30.9798	560	3.0	1.000
4	10	325.2590	12.9908	-2.92	-29.8772	540	3.7	0.999
5	10	311.8766	13.3935	-2.76	-30.9508	558	5.3	0.998
6	12.5	220.3158	14.7742	-2.34	-29.9192	540	4.0	0.996
7*	13	210.8181	14.8316	-2.01	-31.1228	560	3.2	0.994
8	14	184.0869	15.4483	-2.01	-31.0299	560	4.3	0.992
9	11	298.2082	13.3674	-2.87	-29.9141	540	6.1	0.995
10	11	308.4115	13.4294	-2.83	-31.1362	560	4.1	0.999
11	11	320.2009	13.0114	-3.07	-29.9279	540	3.2	1.000
12	11	353.3562	12.6101	-3.28	-30.9299	555	3.2	1.000

Degree-days above 18°C (DD18)

Table S4. Parameters and the results of the model fit for the piecewise function for monthly Degree-days above 18°C.

Region	Month	k	a	b	T_0	β	c	Sigma	R^2
All	1	35	252.4909	21.4798	2.68	0.0000	0	1.4	0.949
	2	35	154.5938	19.8062	2.27	0.0000	-220	1.4	0.940
	3	22	218.9100	20.9740	2.63	26.5492	-450	2.4	0.969
	4	23	262.9456	22.1931	2.91	28.4619	-500	3.1	0.988
	5	23	270.0578	22.1579	2.84	28.6688	-500	4.5	0.993
	6*	21	154.5632	19.7346	2.01	28.8946	-510	4.1	0.998
	7*	22	181.7177	20.5247	1.83	30.7090	-550	3.3	0.999
	8*	22	190.7156	20.6291	1.98	30.7189	-550	4.4	0.998
	9*	24	255.1446	21.7683	2.65	29.2723	-520	5.8	0.994
	10	23	236.9956	21.3134	2.59	28.7088	-500	3.8	0.989
	11	21	159.6297	19.5192	2.33	24.0432	-400	2.5	0.967
	12	21	144.0946	18.9260	2.37	24.2537	-400	1.5	0.955
South west	1	35	194.5122	17.7804	1.09	0.0000	0	0.5	0.997
	2	35	142.9417	19.5130	1.59	0.0000	-220	0.9	0.976
	3	22	83.1831	17.6914	1.45	20.5749	-300	1.8	0.979
	4	23	173.0118	20.0616	1.94	28.4684	-500	2.9	0.991
	5	23	215.0754	20.9080	2.10	28.7549	-500	5.5	0.993
	10	23	226.2045	21.2092	2.13	28.7587	-500	4.6	0.992
	11	18	77.4705	16.6507	1.24	18.6903	-280	7.1	0.966
	12	21.9	148.7235	18.9921	1.82	17.3182	-250	4.2	0.985
The rest	1	35	204.2576	20.8281	2.95	0.0000	0	0.8	0.980
	2	18	71.9182	16.7546	2.22	14.8919	-220	0.7	0.987
	3	20	203.7113	20.7024	2.78	21.9203	-350	1.6	0.987
	4	23	282.6539	22.6475	3.08	28.4613	-500	2.4	0.992
	5	23	272.4801	22.2227	2.91	28.6468	-500	3.8	0.995
	10	23	215.8640	20.7702	2.55	28.6914	-500	2.8	0.994
	11	20	157.7828	19.8833	2.55	20.6859	-330	1.6	0.960
	12	17	63.0808	15.4078	2.11	14.2700	-200	0.8	0.969

* Region specific model is not necessary.

Number of frost-free days (NFFD)

The general function for monthly NFFD (*$NFFD_m$*) is:

$$NFFD_m = \frac{a}{1+e^{-\left(\frac{T_m - T_0}{b}\right)}}$$

where, T_m is the monthly minimum temperature for the *m* month; *a, b and* T_0 are the three parameters to be optimized.

Table S5. Parameters and the results of the model fit for monthly Number of frost-free days.

Month	a	b	T_0	Sigma	R^2
1	31.9203	0.9570	3.82	0.7	0.991
2	29.4221	1.0269	3.80	0.6	0.992
3	31.9966	0.8957	3.60	0.8	0.993
4	30.4145	0.6998	3.13	0.9	0.987
5	31.2379	0.6002	2.78	0.8	0.958
6	30.0053	0.3895	2.24	0.3	0.962
7	30.9517	0.6556	1.23	0.2	0.923
8	30.9461	0.3299	1.71	0.2	0.949
9	30.1120	0.5857	2.72	0.5	0.955
10	31.5968	0.6504	3.23	0.8	0.978
11	30.5354	0.8838	3.47	0.7	0.992
12	31.3262	0.8329	3.63	0.8	0.989

Frost-free period (FFP), the day of the year on which FFP begins (bFFP) and the day of the year on which FFP ends (eFFP)

$bFFP = 352.1358994 + -0.021715653 * Tmin(4)^2 + -3.542187618 * Tmin(6) + 0.020359471 * Tmin(6)^2 - 4.897998097 * TD + 0.033521327 * TD^2 - 2.164862277 * NFFD + 0.006767633 * NFFD^2 - 0.00000929 * NFFD^3 + 0.043516586 * (TD * NFFD) - 0.00000253 * (TD * NFFD)^2$

$eFFP = 243.7752209 + 4.134210825 * Tmin(9) - 0.162876448 * Tmin(9)^2 + 1.248649021 * Tmin(10) + 0.145073612 * Tmin(10)^2 + 0.004319892 * Tmin(11) + -0.005753127 * Tmin(11)^2 - 0.06296471 * NFFD + 0.000399177 * NFFD^2$

FFP = eFFP – bFFP

where Tmin is the monthly minimum temperature, TD is difference between the mean warmest monthly temperature and the mean coldest monthly temperature, and NFFD is the number of frost-free days.

Precipitation as snow (PAS)

The general function for monthly PAS (PAS_m) is

$$PAS_m = \frac{1}{1+e^{-\left(\frac{T_m - T_0}{b}\right)}}$$

Where, T_m is the monthly minimum temperature for the *m* month; *b and* T_0 are the three parameters to be optimized.

Table S6. Parameters and the results of the model fit for monthly precipitation as snow.

Month	b	T_0	Sigma	R^2
1	-4.1625	-2.5114	0.13	0.796
2	-2.6996	-1.7031	0.13	0.804
3	-1.7860	-1.2583	0.07	0.728
4	1.7672	-1.4152	0.05	0.641
5	1.4390	-2.2797	0.01	0.325
6*	1.4390	-2.2797		
7*	2.3201	-2.1302		
8*	3.2012	-1.9808		
9	3.2012	-1.9808	0.01	0.308
10	2.3486	-1.4464	0.03	0.686
11	-1.6709	-1.4617	0.05	0.853
12	-3.0127	-1.5327	0.12	0.823

* No parameters can be determined due to extreme small amount of snow fall in these months. The parameters for May were used for June, the parameters for September were used for August, and the averages between May and September were used for July.

Extreme minimum temperature (EMT) and extreme maximum temperature (EXT)

EMT = -23.02164 + 0.77908 * Tmin(1) + 0.67048 * Tmin(12) + 0.01075 * $TminX^2$ + 0.11565 * TD

EXT = 10.64245 + -1.92005 * Tmax(7) + 0.04816 * $Tmax(7)^2$ + 2.51176 * Tmax(8) - 0.03088 * $Tmax(8)^2$ - 0.01311 * $TmaxX^2$ + 0.33167 * TD - 0.001 * TD^2

where Tmin is the monthly minimum temperature, Tmax is the monthly maximum temperature, TmaxX is the maximum Tmax over the year, and TD is difference between the mean warmest monthly temperature and the mean coldest monthly temperature.

Relative humidity (RH)

Monthly average relative humidity (RH %) is calculated from the monthly maximum and minimum air temperature following [21]:

$$RH = 100 * es(Tmin)/es(avg)$$

$$es(avg) = [es(Tmin) + es(Tmax)]/2$$

where es(Tmin) and es(Tmax) are the saturated vapour pressure (kPa) at the monthly mean minimum and maximum air temperature (°C), respectively, and es(avg) is the monthly average saturation vapour pressure (kPa). The Teten’s equation is used to calculate the saturated vapour pressure (SVP(T) kPa) as a function of temperature (T °C).

$$SVP(T)\ (kPa) = 0.6105 * \exp([17.273 * T]/[T + 237.3])$$

$$\text{For } T => 0^\circ C \quad es(T) = SVP(T)$$

$$\text{For } T < 0^\circ C \quad es(T) = SVP(T) * (1 + [T * 0.01])$$

This method will slightly overestimate the daily average relative humidity in dry environments where the nighttime relative humidity does not approach 100%.
