# Supplementary material for: Locally Downscaled and Spatially Customizable Climate Data for Historical and Future Periods for North America
Source: PLoS One. 2016 Jun 8;11(6):e0156720. doi: 10.1371/journal.pone.0156720 (PMC4898765; doi:10.1371/journal.pone.0156720)
Supplement: S2 Table — (PDF) [file pone.0156720.s003.pdf]

S2 Table. Parameters and the results of the model fit for the piecewise function for monthly Degree-days above 5°C (DD > 5).

Region	Month	k	a	b	T_0	β	c	Sigma	R^2
All	1	12	337.5699	10.0241	3.36	30.0966	-140	7.6	0.986
	2	12	302.8633	10.0060	3.31	28.0429	-140	7.5	0.988
	3	12	363.8180	10.5024	3.44	30.1222	-140	10.3	0.991
	4	12	339.8059	10.3516	3.26	29.4187	-140	7.4	0.997
	5*	12	327.1587	10.0530	2.79	30.1473	-140	3.6	0.999
	6*	13	370.5585	11.1296	3.13	29.9647	-150	2.2	1.000
	7*	15	410.0218	11.6278	3.12	30.7456	-150	1.7	1.000
	8*	15	412.2794	11.6613	3.13	30.7429	-150	1.6	1.000
	9*	13	342.8546	10.5144	2.96	29.7243	-145	2.1	1.000
	10*	12	344.9987	10.2648	3.19	30.4110	-145	5.0	0.999
	11	11	304.7169	9.5882	3.15	29.4263	-140	7.0	0.995
	12	12	341.0866	10.0869	3.29	30.1269	-140	7.0	0.990
West	1	12	299.5106	9.6754	2.58	29.8768	-140	4.3	0.997
	2	12	250.6012	9.2207	2.42	27.9937	-140	5.0	0.997
	3	12	318.4976	10.0133	2.63	30.0353	-140	6.3	0.997
	4	12	308.1068	10.0143	2.75	29.3949	-140	5.9	0.998
	11	12	322.3119	10.2178	2.85	29.3400	-140	5.0	0.998
	12	12	289.4347	9.4137	2.55	30.0054	-140	4.9	0.996
East	1	12	295.5816	8.7948	3.29	30.1798	-140	4.0	0.996
	2	12	283.2021	9.3056	3.44	28.0894	-140	3.9	0.996
	3	12	308.5948	9.1350	3.34	30.1602	-140	6.5	0.996
	4	12	309.8332	9.6150	3.13	29.4255	-140	5.9	0.998
	11	12	309.4319	9.5599	3.26	29.4366	-140	5.0	0.997
	12	12	326.1452	9.5931	3.39	30.1914	-140	3.8	0.997

* Region specific model is not necessary.
