# Supplementary material for: Locally Downscaled and Spatially Customizable Climate Data for Historical and Future Periods for North America
Source: PLoS One. 2016 Jun 8;11(6):e0156720. doi: 10.1371/journal.pone.0156720 (PMC4898765; doi:10.1371/journal.pone.0156720)
Supplement: S3 Table — (PDF) [file pone.0156720.s004.pdf]

S3 Table. Parameters and the results of the model fit for the piecewise function for monthly Degree-days below 18°C (DD < 18).

Month	k	a	b	T_0	β	c	Sigma	R^2
1	11	342.1497	12.7839	-3.19	-30.7428	560	2.9	1.000
2	11	344.9851	12.0296	-3.29	-28.0059	500	2.9	1.000
3	11	325.8230	13.1044	-2.99	-30.9798	560	3.0	1.000
4	10	325.2590	12.9908	-2.92	-29.8772	540	3.7	0.999
5	10	311.8766	13.3935	-2.76	-30.9508	558	5.3	0.998
6	12.5	220.3158	14.7742	-2.34	-29.9192	540	4.0	0.996
7*	13	210.8181	14.8316	-2.01	-31.1228	560	3.2	0.994
8	14	184.0869	15.4483	-2.01	-31.0299	560	4.3	0.992
9	11	298.2082	13.3674	-2.87	-29.9141	540	6.1	0.995
10	11	308.4115	13.4294	-2.83	-31.1362	560	4.1	0.999
11	11	320.2009	13.0114	-3.07	-29.9279	540	3.2	1.000
12	11	353.3562	12.6101	-3.28	-30.9299	555	3.2	1.000
