# Supplementary material for: Locally Downscaled and Spatially Customizable Climate Data for Historical and Future Periods for North America
Source: PLoS One. 2016 Jun 8;11(6):e0156720. doi: 10.1371/journal.pone.0156720 (PMC4898765; doi:10.1371/journal.pone.0156720)
Supplement: S4 Table — (PDF) [file pone.0156720.s005.pdf]

S4 Table. Parameters and the results of the model fit for the piecewise function for monthly Degree-days above 18°C (DD > 18).

Region	Month	k	a	b	T_0	β	c	Sigma	R^2
All	1	35	252.4909	21.4798	2.68	0.0000	0	1.4	0.949
	2	35	154.5938	19.8062	2.27	0.0000	-220	1.4	0.940
	3	22	218.9100	20.9740	2.63	26.5492	-450	2.4	0.969
	4	23	262.9456	22.1931	2.91	28.4619	-500	3.1	0.988
	5	23	270.0578	22.1579	2.84	28.6688	-500	4.5	0.993
	6*	21	154.5632	19.7346	2.01	28.8946	-510	4.1	0.998
	7*	22	181.7177	20.5247	1.83	30.7090	-550	3.3	0.999
	8*	22	190.7156	20.6291	1.98	30.7189	-550	4.4	0.998
	9*	24	255.1446	21.7683	2.65	29.2723	-520	5.8	0.994
	10	23	236.9956	21.3134	2.59	28.7088	-500	3.8	0.989
	11	21	159.6297	19.5192	2.33	24.0432	-400	2.5	0.967
	12	21	144.0946	18.9260	2.37	24.2537	-400	1.5	0.955
South west	1	35	194.5122	17.7804	1.09	0.0000	0	0.5	0.997
	2	35	142.9417	19.5130	1.59	0.0000	-220	0.9	0.976
	3	22	83.1831	17.6914	1.45	20.5749	-300	1.8	0.979
	4	23	173.0118	20.0616	1.94	28.4684	-500	2.9	0.991
	5	23	215.0754	20.9080	2.10	28.7549	-500	5.5	0.993
	10	23	226.2045	21.2092	2.13	28.7587	-500	4.6	0.992
	11	18	77.4705	16.6507	1.24	18.6903	-280	7.1	0.966
	12	21.9	148.7235	18.9921	1.82	17.3182	-250	4.2	0.985
The rest	1	35	204.2576	20.8281	2.95	0.0000	0	0.8	0.980
	2	18	71.9182	16.7546	2.22	14.8919	-220	0.7	0.987
	3	20	203.7113	20.7024	2.78	21.9203	-350	1.6	0.987
	4	23	282.6539	22.6475	3.08	28.4613	-500	2.4	0.992
	5	23	272.4801	22.2227	2.91	28.6468	-500	3.8	0.995
	10	23	215.8640	20.7702	2.55	28.6914	-500	2.8	0.994
	11	20	157.7828	19.8833	2.55	20.6859	-330	1.6	0.960
	12	17	63.0808	15.4078	2.11	14.2700	-200	0.8	0.969

* Region specific model is not necessary.
