# Supplementary material for: Locally Downscaled and Spatially Customizable Climate Data for Historical and Future Periods for North America
Source: PLoS One. 2016 Jun 8;11(6):e0156720. doi: 10.1371/journal.pone.0156720 (PMC4898765; doi:10.1371/journal.pone.0156720)
Supplement: S5 Table — (PDF) [file pone.0156720.s006.pdf]

S5 Table. Parameters and the results of the model fit for monthly Number of frost-free days (NFFD).

Month	a	b	T_0	Sigma	R^2
1	31.9203	0.9570	3.82	0.7	0.991
2	29.4221	1.0269	3.80	0.6	0.992
3	31.9966	0.8957	3.60	0.8	0.993
4	30.4145	0.6998	3.13	0.9	0.987
5	31.2379	0.6002	2.78	0.8	0.958
6	30.0053	0.3895	2.24	0.3	0.962
7	30.9517	0.6556	1.23	0.2	0.923
8	30.9461	0.3299	1.71	0.2	0.949
9	30.1120	0.5857	2.72	0.5	0.955
10	31.5968	0.6504	3.23	0.8	0.978
11	30.5354	0.8838	3.47	0.7	0.992
12	31.3262	0.8329	3.63	0.8	0.989
