# Supplementary material for: Locally Downscaled and Spatially Customizable Climate Data for Historical and Future Periods for North America
Source: PLoS One. 2016 Jun 8;11(6):e0156720. doi: 10.1371/journal.pone.0156720 (PMC4898765; doi:10.1371/journal.pone.0156720)
Supplement: S6 Table — (PDF) [file pone.0156720.s007.pdf]

S6 Table. Parameters and the results of the model fit for monthly precipitation as snow (PAS).

Month	b	T_0	Sigma	R^2
1	-4.1625	-2.5114	0.13	0.796
2	-2.6996	-1.7031	0.13	0.804
3	-1.7860	-1.2583	0.07	0.728
4	1.7672	-1.4152	0.05	0.641
5	1.4390	-2.2797	0.01	0.325
6*	1.4390	-2.2797		
7*	2.3201	-2.1302		
8*	3.2012	-1.9808		
9	3.2012	-1.9808	0.01	0.308
10	2.3486	-1.4464	0.03	0.686
11	-1.6709	-1.4617	0.05	0.853
12	-3.0127	-1.5327	0.12	0.823

* No parameters can be determined due to extreme small amount of snow fall in these months. The parameters for May were used for June, the parameters for September were used for August, and the averages between May and September were used for July.

Extreme minimum temperature (EMT) and extreme maximum temperature (EXT)

EMT = -23.02164 + 0.77908 * Tmin(1) + 0.67048 * Tmin(12) + 0.01075 * $TminX^2$ + 0.11565 * TD

EXT = 10.64245 + -1.92005 * Tmax(7) + 0.04816 * $Tmax(7)^2$ + 2.51176 * Tmax(8) - 0.03088 * $Tmax(8)^2$ - 0.01311 * $TmaxX^2$ + 0.33167 * TD - 0.001 * TD^2

where Tmin is the monthly minimum temperature, Tmax is the monthly maximum temperature, TmaxX is the maximum Tmax over the year, and TD is difference between the mean warmest monthly temperature and the mean coldest monthly temperature.

Relative humidity (RH)

Monthly average relative humidity (RH %) is calculated from the monthly maximum and minimum air temperature following [21]:

$$RH = 100*es(Tmin)/es(avg)$$

$$es(avg) = [es(Tmin)+ es(Tmax)]/2$$

where es(Tmin) and es(Tmax) are the saturated vapour pressure (kPa) at the monthly mean minimum and maximum air temperature (°C), respectively, and es(avg) is the monthly average saturation vapour pressure (kPa). The Teten's equation is used to calculate the saturated vapour pressure (SVP(T) kPa) as a function of temperature (T °C).

$$SVP(T)\ (kPa) = 0.6105*\exp([17.273*T]/[T+237.3])$$

For T=> 0°C   es(T) = SVP(T)

For T<0°C   es(T) = SVP(T)*(1 + [T*0.01])

This method will slightly overestimate the daily average relative humidity in dry environments where the nighttime relative humidity does not approach 100%.
